# 24-month decline of non-invasive liver fibrosis markers in HCV-mono and HCV/HIV coinfection after direct-acting antiviral therapy

**DOI:** 10.1038/s41598-022-07548-y

**Published:** 2022-03-09

**Authors:** Laura Pérez-Is, Julio Collazos, Belén de la Fuente, Luis Morano, Maria Rivas-Carmenado, Manuel Rodriguez, Adolfo Romero-Favela, Galilea de Jesús Fonseca–González, Santiago Melón, Eulalia Valle-Garay, Víctor Asensi

**Affiliations:** 1 Biochemistry and Molecular Biology Oviedo, School of Medicine, University of Oviedo, Oviedo, Spain; 2 Infectious Diseases Unit, Hospital de Galdácano, Galdakao, Spain; 3 Infectious Diseases Unit, Hospital de Cabueñes, Gijón, Spain; 4 Infectious Diseases Unit, Santiago de Compostela University Medical School, University Hospital Álvaro Cunqueiro, Vigo, Spain; 5 Infectious Diseases-HIV Unit, Hospital Universitario Central de Asturias, School of Medicine, University of Oviedo, Oviedo, Spain; 6 Liver Unit, Division of Gastroenterogy and Hepatology, Hospital Universitario Central de Asturias, University of Oviedo, Oviedo, Spain; 7 Unidad Académica de Ingeniería en Biotecnología, Universidad Politécnica de Sinaloa, Mazatlán, Mexico; 8 Virology Section, Hospital Universitario Central de Asturias, School of Medicine, University of Oviedo, Avda Romas/ S/N, 33011 Oviedo, Spain; 9 Group of Translational Research in Infectious Diseases, Instituto de Investigación Sanitaria del Principado de Asturias (ISPA), Oviedo, Spain

**Keywords:** Biomarkers, Diseases, Infectious diseases

## Abstract

Long term liver fibrosis (LF) changes and their best -monitoring non-invasive markers (NILFM) after effective anti-HCV DAA therapy are little- known. Matrix-metalloproteases (MMPs) and their tissue-inhibitors (TIMPs) are pivotal in liver inflammation repair. Their plasma levels might assess long-term LF changes after therapy. Overall 374 HCV-infected adult patients, 214 HCV-HIV coinfected, were followed-up for 24 months after starting DAA. LF was assessed by transient elastometry (TE), biochemical indexes (APRI, Forns, FIB-4) and, in 61 individuals, by MMPs and TIMP-1 plasma levels. Several MMPs and TIMP-1 SNPs were genotyped in 319 patients. TE was better than biochemical indexes for early and long-term LF monitoring. MMPs-2,-8,-9 and-TIMP-1 levels and TE displayed parallel declining curves although only TIMP-1 correlated with TE (*P* = 0.006) and biochemical indexes (*P* < 0.02). HCV monoinfected had significantly higher baseline NILFM and TIMP-1 plasma values, but lower MMPs levels than coinfected patients. No differences in NILFM course were observed between mono-and coinfected or between different DAA regimens. Only the *MMP-2 (-1306 C/T)* variant *TT* genotype associated with higher values of NILFM NILFM decline extends 24 months after therapy. TE and TIMP1 are reliable LF-monitoring tools. NILFM courses were similar in mono-and coinfected patients, DAA regimens type did not influence NILFM course.

## Introduction

Hepatitis C virus (HCV) infection, a major cause of progressive liver fibrosis (LF), leads to cirrhosis, and eventually to hepatocellular carcinoma^1^. Direct-acting antiviral agents (DAA) show > 90% efficacy against HCV^2^. LF dynamics at long term after DAA exposition remain elusive due to the lack of extended follow-up studies^3–7^. LF curve decreases quickly after starting DAA therapy and then a slow decline or even a plateau follows. LF quick decrease, common but not invariable, is due to early resolution of liver inflammation^8^. Other unknown mechanisms could lie behind the slowly extended LF regression after DAA and, therefore, prolonged monitoring of LF changes after DAA treatment is needed.

Long term LF evaluation has several limitations. Liver biopsy is the reference standard for fibrosis assessment. However, biopsy is invasive, cannot be frequently repeated and its interpretation has recognized limitations^9^. Thus transient elastometry (TE), and noninvasive biochemical biomarkers of LF (APRI, Forns, FIB-4), have been successfully used to evaluate LF in HCV monoinfected and HCV-HIV coinfected patients^10–18^. Nevertheless, whilst noninvasive biochemical indexes and TE correlate with LF biopsy findings, liver inflammation reversal temporarily limits the former’s value. TE detects extended LF changes but does not inform of its cause. Some authors consider TE the best tool for monitoring fibrosis regression following cure in DAA-treated HCV-HIV infected patients^19,20^. However the routine use of non-invasive scores and liver stiffness measurements (LSM) by TE and other elastography methods following antiviral therapy in HCV-infected patients is currently not recommended to detect fibrosis by the European Association for the Study of the Liver (EASL) Clinical Practice Guidelines^21^.

Therefore, new tools to assess LF at long term after DAA therapy are needed, particularly, if linked to the pathogenic mechanisms underlying LF generation and regression.

Matrix metalloproteinases (MMPs), zinc-dependent endopeptidases, play key biological roles including inflammation and tissue repair. MMPs are controlled by specific tissue inhibitors (TIMPs), enhancing extracellular matrix (ECM) synthesis and reducing connective tissue proteins degradation^22^. A MMPs/TIMPs imbalance favoring MMPs or reducing TIMPs leads to LF^23,24^. MMP-2 and MMP-9 show increased activity in rat liver and in chronic HCV-HIV mono and coinfected patients´ plasma^25,26^. Other MMPs such as MMP1, MMP13 and TIMP1 are also involved in LF secondary to chronic HCV infection^27–30^. MMPs activity is regulated at gene expression level and by activation of latent pro-MMPs to active MMPs^23,24^. MMPs polymorphisms (SNPs) might modify gene expression and MMPs plasma levels, and indirectly influence LF^31–35^. MMPs plasma levels are higher in HIV-infected naïve compared to HCV monoinfected patients^36–38^, while effective antiretroviral therapy normalizes their plasma MMPs levels^37^. HCV Core and NS5A proteins increase intrahepatic expression of COX, MMP-2 and MMP-9 in “in vitro” transfection assays. This effect is mediated by different transcription activation factors leading to intracellular Ca^++^ changes inducing *COX-2* gene expression and peroxisome proliferator-activated receptor (PPAR)-α ligands activation^39,40^.

We hypothesized that LF reversal after DAA treatment lengthen. This LF regression might be due to ECM remodeling, rather than reduced inflammation, might be expressed as decreasing plasma circulating MMPs levels and might differ with the selected DAA regimen. Few studies have focused on this idea, but their small size, short-follow-up, lack of DAA regimens comparison or MMPs plasma assessment or SNPs genotyping limit their results^30,41^.

The aim of this study was to evaluate the long-term course of LF, as measured by noninvasive markers, in HCV mono- and HCV/HIV coinfected patients following DAA therapy, and to identify the factors independently associated with non-invasive liver fibrosis markers (NILFM) decline and HCV mono and HCV/HIV coinfection. Secondarily, we also evaluated the role of different MMPs SNPs on liver fibrosis assessed by NILFM, before and after DAA therapy, and, in a subset of patients, the behavior of MMP 2,-8,-9 and-TIMP-1 plasma levels. To this end, a large cohort of HCV mono- and HCV/HIV coinfected patients was prospectively followed-up at regular intervals for 24 months.

## Results

A total of 374 patients, 160 HCV-monoinfected and 214 HCV/HIV coinfected, were included in the study. Overall, the mean age was 50.8 years (95% CI 50.0–51.6), and 70.6% were men. Among HIV/HCV co-infected patients the HIV viral load was undetectable in 74.6% of the patients at baseline and HIV undetectability increased to 88.3% at the 24th month measurement. The mean CD4 counts were 614.1 cells/µL (95% CI 566.0–662.3) at baseline, and 626.60 cells/µL (95% CI 575.89–677.30) at the end of follow-up. Patients were treated with diverse DAA regimens and were evaluated at baseline and at the 1st, 3rd, 6th, 12th, and 24th month afterwards, although 85 patients did not complete the final evaluation.

Table 1 shows the demographic, HCV, laboratory and NILFM parameters of the patients as a whole, as well as those of the mono- and coinfected patients separately. It can be appreciated that there were statistically significant differences between mono- and coinfected patients in HCV genotype, leukocyte counts, ALT, total proteins, urea, creatinine, and HDL-cholesterol.

**Table 1:** Demographic, HCV-related, laboratory and NILFM parameters in mono- and coinfected patients.

		All (n = 374)	Monoinfected (n = 160)	Coinfected (n = 214)	P value
**Demography**					
Gender	Male	264 (70.6%)	116 72.5(%)	148 (69.2%)	0.5
	Female	110 (29.4%)	44 (27.5%)	66 (30.8%)	
Age	Years	50.81 (50.02–51.59)	51.33 (49.96–52.70)	50.42 (49.50–51.33)	0.3
**HCV-related parameters**					
HCV viral load at baseline	Log copies/mL	5.896 (5.767–6.025)	5.880 (5.726–6.034)	5.908 (5.713–6.102)	0.8
HCV genotype	1	205 (75.6%)	81 (81.8%)	124 (72.1%)	0.035
	2	2 (0.7%)	1 (1.0%)	1 (0.6%)	
	3	41 (15.1%)	15 (15.2%)	26 (15.1%)	
	4	23 (8.5%)	2 (2.0%)	21 (12.2%)	
**HCV treatment**					
Time on DAA therapy	Weeks	13.09 (12.66–13.52)	12.60 (12.04–13.17)	13.45 (12.84–14.07)	0.054
Sofosbuvir	Yes	325 (86.9%)	137 (85.6%)	188 (87.9%)	0.53
	No	49 (13.1%)	23 (14.4%)	26 (12.1%)	
Drug combinations	Sofosb + Velpatasvir	149 (39.8%)	62 (38.8%)	87 (40.7%)	0.7
	Sofosb + Ledipasvir	141 (37.7%)	59 (36.9%)	82 (38.3%)	
	Other combinations	84 (22.5%)	39 (24.4)	45 (21.0%)	
HCV eradicated	Yes	363 (98.4%)	153 (98.1%)	210 (98.6%)	0.7
	No	6 (1.6%)	3 (1.9%)	3 (1.4%)	
**Laboratory blood parameters at baseline**					
Hemoglobin	g/dL	14.72 (14.55–14.89)	14.84 (14.61–15.07)	14.63 (14.39–14.87)	0.2
Leukocytes	cells/ μL	6612 (6366–6859)	6916 (6557–7276)	6383 (6047–6719)	0.035
Platelets	× 1000/μL	178.2 (171.0–185.5)	183.5 (171.6–195.3)	174.3 (165.3–183.4)	0.2
Aspartate aminotransferase	U/L	69.2 (62.8–75.6)	70.9 (62.0–79.9)	67.6 (58.4–76.9)	0.6
Alanine aminotransferase	U/L	76.8 (69.4–84.3)	88.0 (74.7–101.3)	68.4 (60.1–76.7)	0.01
Alkaline phosphatase	U/L	90.7 (87.1–94.4)	90.4 (84.6–96.1)	91.0 (86.2–95.8)	0.9
γ-glutamyl transferase	U/L	145.6 (112.3–178.8)	128.7 (104.1–153.4)	159.3 (102.2–216.5)	0.4
Total bilirubin	mg/dL	0.959 (0.909–1.009)	0.943 (0.864–1.022)	0.971 (0.905–1.037)	0.6
Total proteins	g/dL	7.617 (7.520–7.714)	7.428 (7.306–7.550)	7.705 (7.576–7.835)	0.008
Albumin	g/dL	4.279 (4.230–4.329)	4.320 (4.246–4.395)	4.247 (4.181–4.313)	0.14
Fibrinogen	mg/dL	366.9 (352.9–381.0)	354.4 (334.4–374.4)	374.0 (355.1–393.0)	0.19
INR		1.066 (1.041–1.090)	1.084 (1.037–1.131)	1.050 (1.028–1.072)	0.18
Glucose	mg/dL	104.8 (100.3–109.2)	108.0 (99.6–116.4)	102.7 (97.7–107.6)	0.3
Urea	mg/dL	34.8 (33.5–36.0)	33.2 (31.3–35.0)	35.8 (34.1–37.5)	0.045
Creatinine	mg/dL	0.849 (0.829–0.870)	0.796 (0.767–0.826)	0.890 (0.863–0.917)	< 0.0001
Total cholesterol	mg/dL	169.5 (165.2–173.8)	167.9 (161.3–174.5)	170.8 (165.0–176.5)	0.5
HDL cholesterol	mg/dL	49.6 (47.5–51.8)	52.2 (48.6–55.7)	47.6 (45.1–50.2)	0.037
LDL cholesterol	mg/dL	95.2 (91.0–99.4)	92.7 (86.8–98.6)	97.2 (91.2–103.2)	0.3
HIV viral load	log copies/mL	–	–	0.677 (0.476–0.878)	–
CD4 lymphocytes	cells/μL	–	–	614.1 (566.0–662.3)	–
CD4 lymphocytes	%	–	–	30.73 (29.13–32.33)	–
**Fibrosis parameters at baseline**					
Transient elastometry	kPa	10.42 (9.69–11.22)	11.73 (10.44–13.17)	9.55 (8.70–10.49)	0.006
APRI index		0.841 (0.678–1.044)	0.973 (0.839–1.129)	0.948 (0.828–1.085)	0.8
Forns index		6.030 (5.500–6.611)	6.050 (5.700–6.421)	6.186 (5.869–6.521)	0.6
FIB-4 index		1.927 (1.602–2.317)	2.086 (1.859–2.341)	2.142 (1.922–2.387)	0.7
Degree of liver fibrosis (measured by TE)	F0-F1	125 (33.5%)	41 (25.8%)	84 (39.3%)	0.03
	F2	76 (20.4%)	36 (22.6%)	40 (18.7%)	
	F3	61 (16.4%)	25 (15.7%)	36 (16.8%)	
	F4	111 (29.8%)	57 (35.8%)	54 (25.2%)	
**Change in fibrosis parameters after 24 months**					
Absolute change in TE^a^	kPa	− 5.29 (− 6.76, − 3.81)	− 6.876 (− 9.49, − 4.26)	− 4.33 (− 6.10, − 2.56)	0.1
Absolute change in APRI^a^		− 1.13 (− 1.40, − 0.86)	− 1.28 (− 1.65, − 0.92)	− 0.99 (− 1.40, − 0.59)	0.3
Absolute change in Forns		− 1.32 (− 1.49, − 1.14)	− 1.26 (− 1.58, − 0.93)	− 1.36 (− 1.56, − 1.16)	0.6
Absolute change in FIB-4^a^		− 1.03 (− 1.33, − 0.72)	− 1.26 (− 1.72, − 0.79)	− 0.83 (− 1.23, − 0.43)	0.16
Relative improvement in TE^b^	%	28.03% (23.5–32.5)	35.4% (28.3–42.6)	23.6% (17.8–29.3)	0.01
Relative improv. in APRI^b^	%	44.2% (30.9–57.5)	52% (35.4–68.6)	37.2% (16.5–57.8)	0.3
Relative improv. in Forns^b^	%	19.1% (16.2–21.9)	17.2% (12.0–22.4)	20.4% (17.2–23.7)	0.3
Relative improv. in FIB-4^b^	%	19.0% (11.1–27.0)	21.6% (7.9–35.3)	16.9% (7.6–26.1)	0.6

Values are expressed as mean (95% CI) or % as appropriate.

DAA denotes direct acting antivirals, HCV hepatitis C virus, HIV human immunodeficiency virus, HDL high density lipoproteins, INR international normalized ratio, LDL low density lipoproteins, NILFM non-invasive liver fibrosis markers, TE transient elastometry.

^a^Difference and ^b^ratio between 24-month and baseline intrasubject measurements.

Regarding NILFM, monoinfected patients had significantly higher degrees of fibrosis at baseline, as evaluated by TE, and also experienced higher degrees of improvement at 24 months following anti-HCV-therapy, differences that were not observed in the other liver fibrosis non-invasive indexes evaluated.

A logistic regression model was elaborated using the variables with a *P* value < 0.1 in the univariate analyses to identify the factors independently associated with mono/coinfection. This model, which adequately fitted the data according to the Hosmer–Lemeshow goodness-of-fit test, revealed that lower baseline measurements of TE (OR 0.901, 95% CI 0.846–0.960, *P* = 0.001), higher serum total proteins (OR 6.194, 95% CI 1.892–20.275, *P* = 0.003), and lower serum HDL-cholesterol (OR 0.959, 95% CI 0.930–0.989, *P* = 0.008) were independently predictive of HIV-HCV-coinfection.

### Course of NILFM following treatment

**Figure 1:**
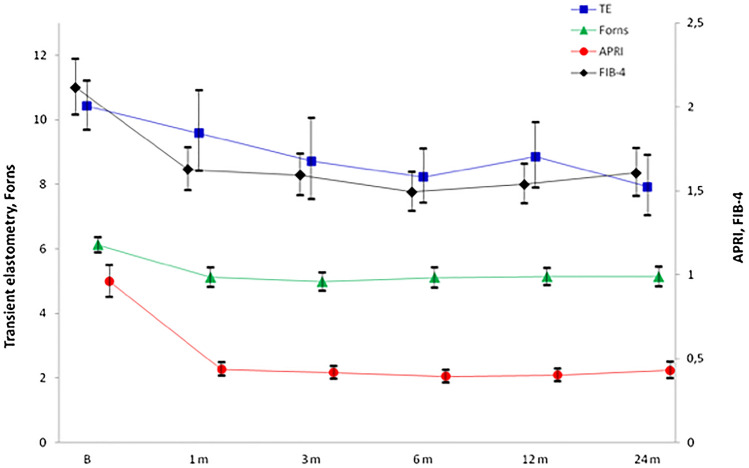
Course of the transient elastometry, APRI, Forns and FIB-4 indexes over time (mean, 95% CI).

Figure 1 depicts the evolution over time of the four NILFM evaluated at each time-point. The curves show that TE measurements declined quite steadily until the end of follow-up. On the contrary, the other three indexes experienced a marked decline during the first month, reaching a plateau afterwards. The paired, intra-subject comparison of baseline vs 24-month values yielded statistically significant decreases for all indexes (*P* < 0.0001 for each).

**Figure 2:**
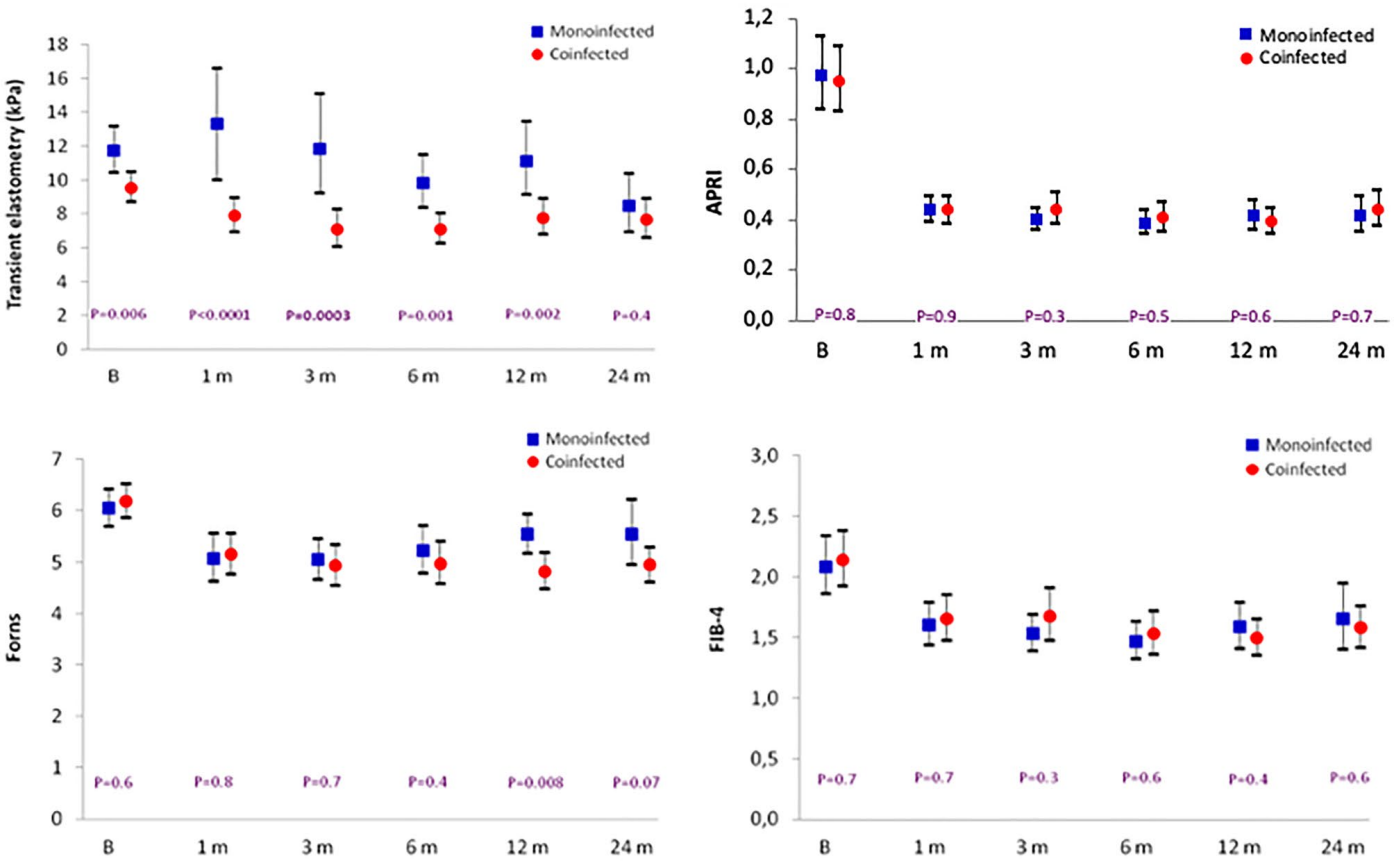
Course over time of the four fibrosis indexes in mono- and coinfected patients (mean, 95% CI).

Figure 2 shows the comparative course over time of the four non-invasive liver fibrosis indexes in mono- and coinfected patients. The degree of fibrosis at each time point was very similar in both groups of patients for APRI, Forns, and FIB-4 indexes, but not for TE, as monoinfected individuals had significantly higher values than coinfected patients in all but the 24-month measurements. Therefore, only TE discriminates between the two patient groups.

**Figure 3:**
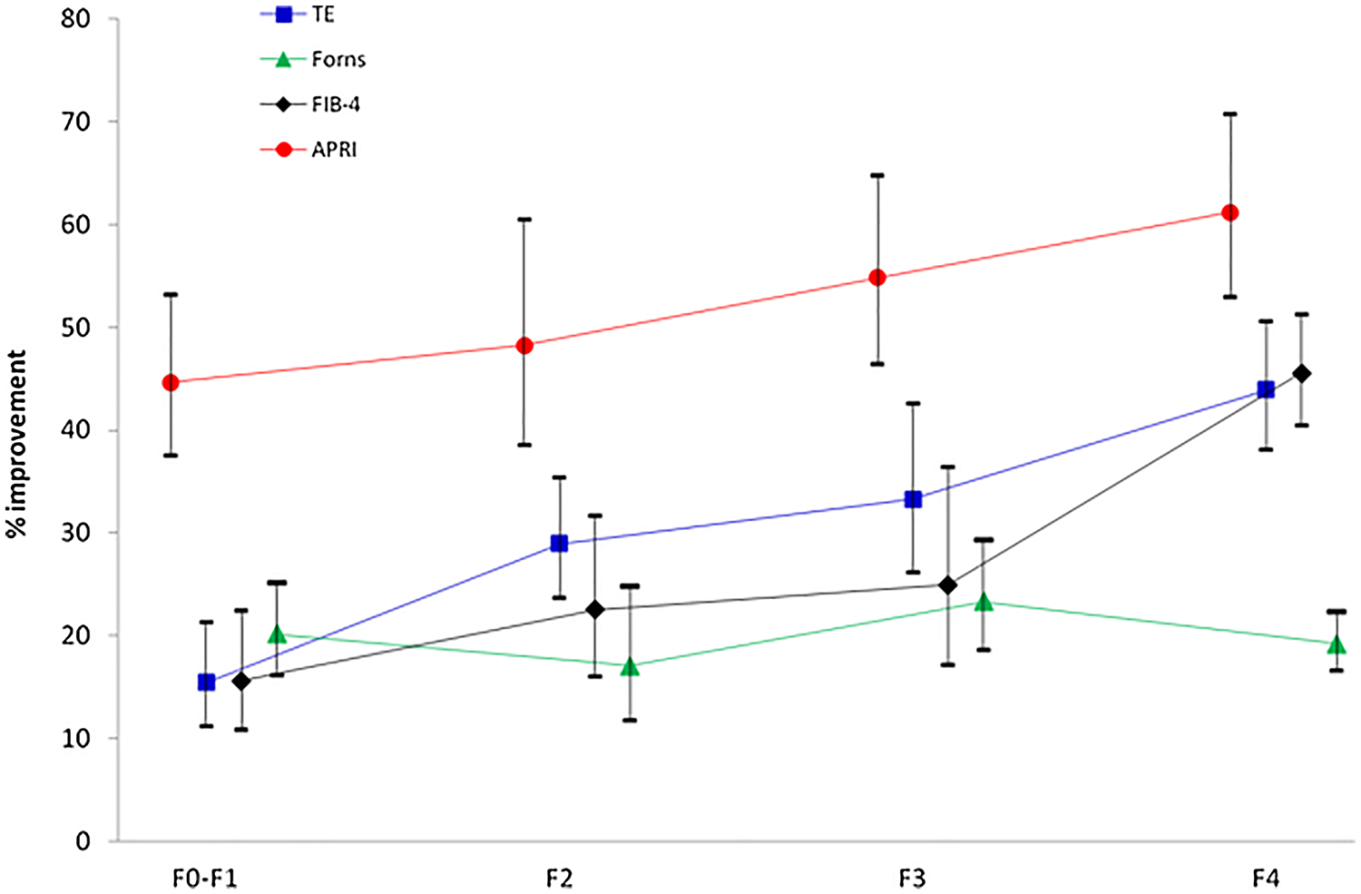
Improvement in the 24 month vs baseline measurements of non-invasive liver fibrosis markers in each baseline fibrosis stage (mean, 95% CI).

Figure 3 displays the proportional improvement in non-invasive liver fibrosis indexes of the 24-month vs the baseline measurements, according to the baseline fibrosis stage. The figure reveals that the more advanced the initial fibrosis stage, the greater intra-subject improvement in fibrosis following therapy for TE (*P* < 0.0001), FIB-4 (*P* < 0.0001), APRI (*P* = 0.025), but not for Forns (*P* = 0.5). No significant differences in improvement existed between mono- and co-infected patients according to the initial fibrosis stage (data not shown).

Multivariate analyses revealed that the strongest predictor for both absolute and relative improvements in NILFM at 24 months as compared to baseline was a more advanced initial fibrosis stage (*P* < 0.0001). The other baseline factors significantly associated with these endpoints were higher gamma-glutamyl transferase GGT levels (*P* = 0.006) for absolute, and higher aspartate aminotransferase AST levels (*P* = 0.008) for relative improvements in fibrosis. These models accounted for 40.2% and 22.4% of the total absolute and relative variability in the improvement, respectively.

### HCV treatment

We also compared the evolution of NILFM depending on the presence or absence of sofosbuvir in the DAA regimen. Patients treated with sofosbuvir had higher degrees of fibrosis at baseline than those receiving other regimens in each of the four fibrosis parameters evaluated: TE 10.75 kPa (95% CI 9.92–11.65) vs. 8.52 (7.22–10.05), respectively, *P* = 0.03; APRI 1.010 (0.906–1.125) vs. 0.717 (0.560–0.918), *P* = 0.02; Forns 6.25 (5.98–6.53) vs. 5.50 (5.06–5.98), *P* = 0.02, and FIB-4 2.203 (2.019–2.403) vs. 1.678 (1.421–1.981), *P* = 0.01.

**Figure 4:**
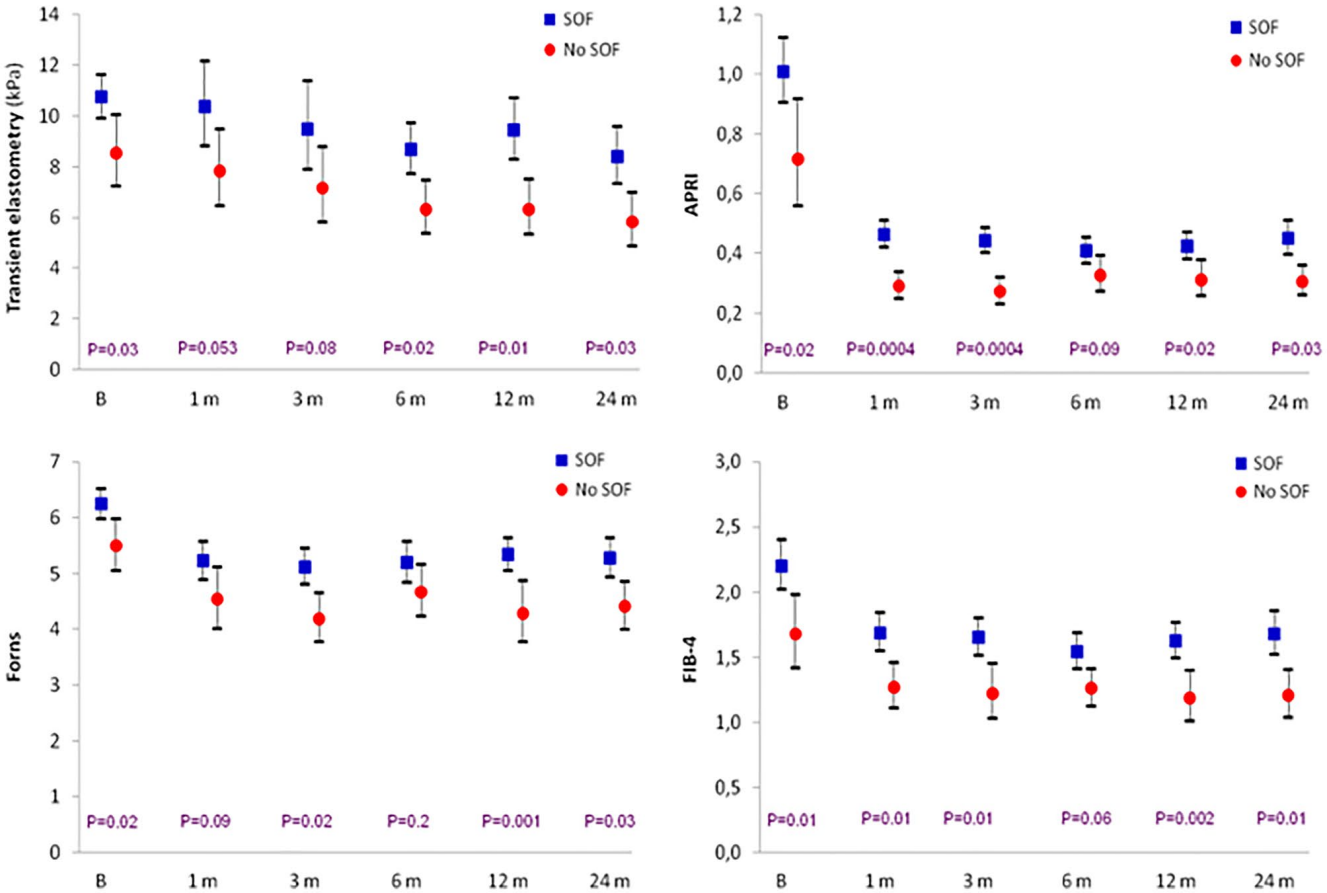
Course over time of the four fibrosis indexes in patients treated or not with sofosbuvir regimens (mean, 95% CI).

The follow-up evaluations revealed that the NILFM curves over time of sofosbuvir and non-sofosbuvir regimens were remarkably parallel (Fig. 4), maintaining, therefore, the initial differences, and evidencing that the fibrosis response was the same in both groups.

### Matrix metalloproteinases and tissue inhibitors

A subset of 61 patients (30 monoinfected and 31 coinfected) underwent sequential measurements of plasma MMP-2, MMP-8, MMP-9 and TIMP-1.

Table 2 describes the correlations among MMPs, TIMP-1 and the four fibrosis indexes. There were highly significant correlations among the diverse MMPs, as well as among the different fibrosis indexes among them, but no MMP correlated with any of the fibrosis indexes. On the contrary, TIMP-1 significantly correlated with each of the four fibrosis parameters.

**Table 2:** Correlations between MMPs, TIMP-1 and fibrosis parameters.

	MMP-8	MMP-9	TIMP-1	TE	APRI	Forns	FIB-4
MMP-2	0.34 (0.008)	0.51 (< 0.0001)	0.21 (0.11)	− 0.08 (0.5)	− 0.02 (0.9)	0.10 (0.5)	0.05 (0.7)
MMP-8		0.78 (< 0.0001)	− 0.11 (0.4)	0.03 (0.8)	− 0.02 (0.9)	0.06 (0.7)	0.03 (0.8)
MMP-9			− 0.11 (0.4)	− 0.14 (0.3)	0.04 (0.8)	0.10 (0.5)	0.01 (0.9)
TIMP-1				0.35 (0.006)	0.31 (0.02)	0.40 (0.006)	0.42 (0.001)
TE					0.62 (< 0.0001)	0.60 (< 0.0001)	0.64 (< 0.0001)
APRI						0.74 (< 0.0001)	0.90 (< 0.0001)
Forns							0.82 (< 0.0001)

r (P value).

TE denotes transient elastometry.

**Figure 5:**
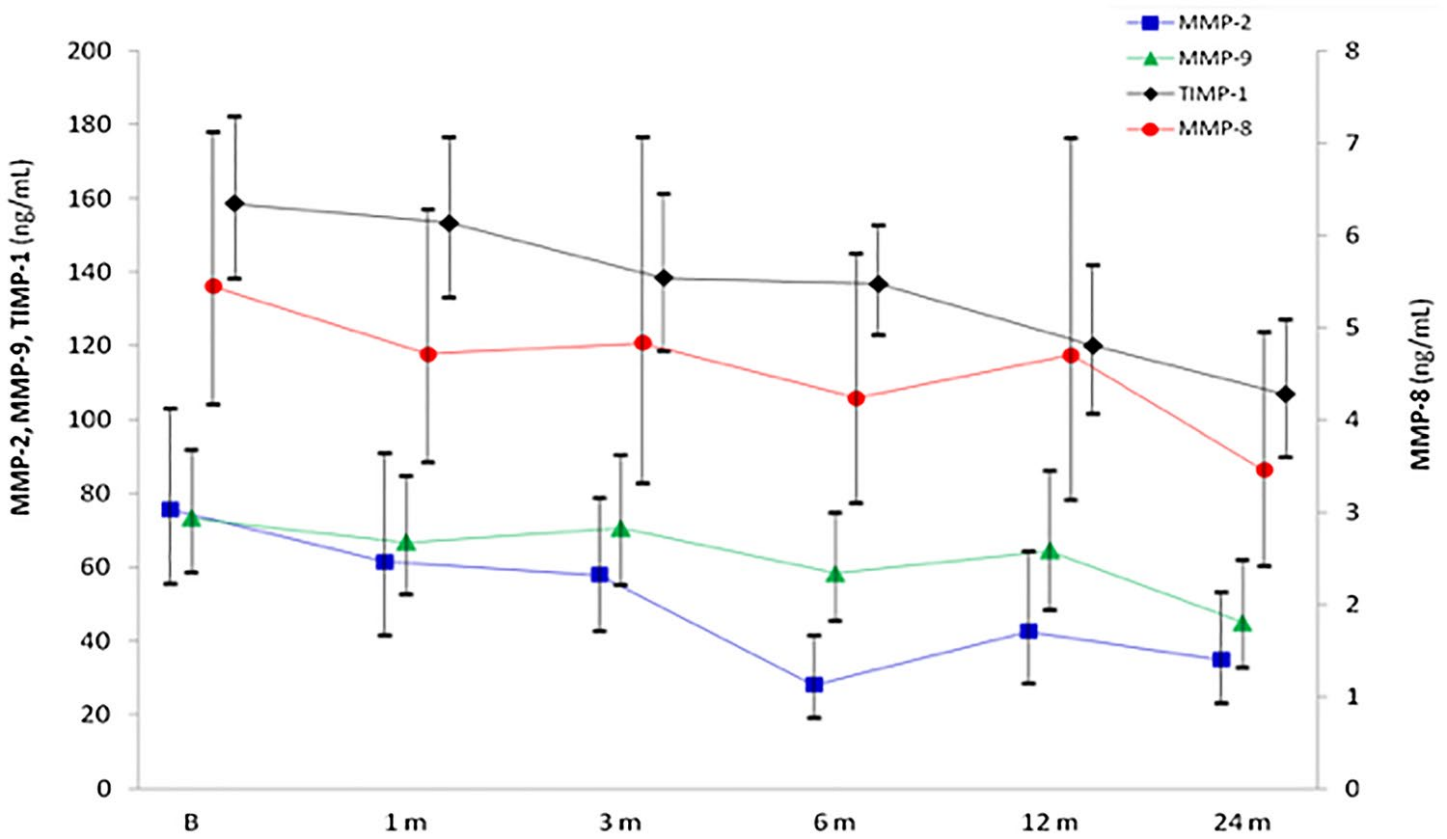
Course over time of MMPs and TIMP-1 (mean, 95% CI).

Figure 5 shows the course over time of MMP-2, -8, -9 and TIMP-1. All of the 4 curves experienced a downward progression. The paired, intra-individual comparison of baseline vs 24-month values revealed statistically significant decreases of MMP-2 (*P* = 0.02), MMP-8 (*P* = 0.009), MMP-9 (*P* = 0.0004) and TIMP-1 (*P* = 0.0001).

**Figure 6:**
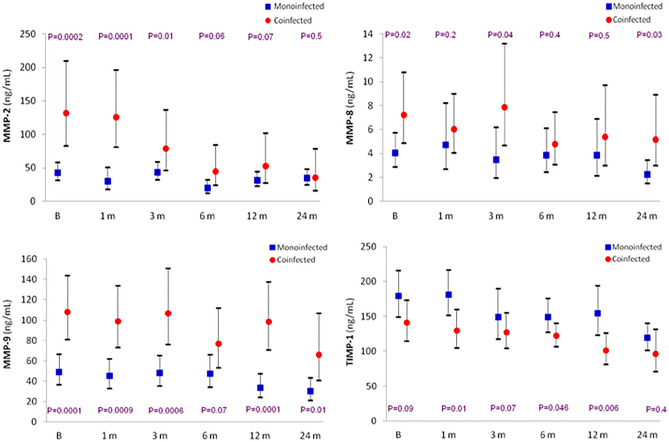
Course over time of MMP-2, MMP-8, MMP-9 and TIMP-1 in mono- and coinfected patients (mean, 95% CI).

Figure 6 depicts the course over time of the three MMPs and TIMP-1 in monoinfected and coinfected patients. Coinfected individuals had substantially higher levels at baseline of each MMP than monoinfected patients, but lower levels in the case of the inhibitor TIMP-1, an opposite pattern that persisted during the 24 months of follow-up.

### Single nucleotide polymorphisms

There were no significant differences among the different genotypes of the SNPs studied in 319 patients according to the mono- or coinfected status of the patients: *MMP-2 (-1306C/T)* (*P* = 0.7), *MMP-2 (-735C/T)* (*P* = 0.4), *MMP-8 (-799C/T )* (*P* = 0.3), *MMP-13 (- 77A/G)* (*P* = 0.4), and *TIMP-1 (9830 T/G)* (*P* = 0.6).

Most of the SNPs evaluated did not show any significant association with the diverse non-invasive liver fibrosis parameters. However, the mutant homozygous *TT* genotype of the *MMP-2 (-1306C/T)* SNP was associated with somewhat higher degrees of NILFM, both at baseline and during the follow-up, whereas the wild *CC* and heterozygous *CT* genotypes had almost identical degrees of fibrosis (supplementary Fig. S1 online). Nevertheless, the reduced number of patients with the mutant *TT* genotype precluded the detection of statistically significant differences, with the exception of the 6-month measurement, at which time point such differences were observed in each of the four fibrosis parameters: TE (*P* = 0.006), APRI (*P* = 0.001), Forns (*P* = 0.04) and FIB-4 (*P* = 0.01).

## Discussion

Our study showed that NILFM decline is a continuous process lasting for at least the 24 months of follow-up after starting DAA therapy. TE was superior to noninvasive biochemical biomarkers (APRI, Forns and FIB-4), which only identified an early LF decrease during the first month after the onset of DAA treatment, reaching since then a plateau in the LF curve that persisted during the remaining follow-up period. Therefore, these biomarkers are not useful for the long-term evaluation of LF following therapy. Plasma MMPs-2, -8, -9 and-TIMP-1 levels experienced a progressive decline in parallel to that of TE curves although only TIMP-1 correlated with TE and biochemical indexes. Therefore TIMP-1 is a useful monitoring tool for long term LF regression, assessed by NILFM, after DAA.

Our study also shows that HCV-monoinfected patients had higher NILFM at baseline than HCV-HIV-coinfected, as evidenced by TE, differences that were maintained at least during one year. On the contrary, LF evaluated by the three biochemical indexes was very similar in both groups at baseline and during follow-up, as a result of the similarity of the two groups in the laboratory parameters in which these indexes are based. Therefore, these biomarkers are not useful for evaluating LF differences in mono- and coinfected patients.

Other authors have also observed higher LF in monoinfected than in coinfected patients at baseline^13^. Taking into account that HCV infection is silent for many years, these differences may be at least partially explained for an earlier detection of HCV infection in coinfected patients, because the controls and medical care required for their HIV infection allow an inmediate suspicion and subsequent diagnosis, as both viruses share the same routes of transmission.

On the other hand, patients with higher LF at baseline experienced greater LF improvements after DAA therapy, as evidenced by TE and other indexes. Besides fibrosis stage at baseline, higher levels of certain liver function tests, such as gamma glutamyl transferase and aspartate aminotransferase, were also independently associated with greater improvements in LF at 24 months as compared to baseline.

Regarding the different DAA therapies, each of the four noninvasive methods of LF evaluation evidenced that patients exposed to sofosbuvir-based regimens had higher LF at baseline than those non-exposed, which reflects the clinicians’ preferences for sofosbuvir in the treatment of patients with advanced fibrosis. However, all four indexes also evidenced that the course over time was strongly parallel in the sofosbuvir and non-sofosbuvir groups, indicating that sofosbuvir-based regimens are not superior to others in LF reduction, as neither was in HCV eradication rates. The high efficacy of the diverse DAA regimens, and the impracticality of the comparison of individual drugs because of their combined use, justify the similar decrements in fibrosis that we observed.

Likewise, no differences between HCV-mono or HCV-HIV-coinfected patients regarding LF were observed in carriers of the different MMPs and TIMP-1 SNPs genotyped. However, variant *T* allele homozygous carriers of the *MMP-2 (-1306 C/T, rs 243865)* SNP had somewhat more fibrosis than other genotypes carriers, with almost identical fibrosis, both at baseline and during follow-up. These discrete differences were observed in TE, but also in the other fibrosis indexes independent of TE, suggesting that the differences exist and that this uncommon genotype may predispose to greater degrees of fibrosis in HCV infection. Similarly, we did not find any association of LF with other MMPs SNPs genotypes in another study without *MMP-2 rs 243865* SNP genotyping^38^. Interestingly this *MMP-2 rs 243,865* SNP has been associated with central obesity and non-alcoholic fat liver disease and with increased risk of cirrhotic hepatopulmonary syndrome in Chinese patients. However the carriage of the variant *TT* genotype of this *MMP-2* SNP decreased the risk for both hepatic complications^46,47^.

Although other studies have dealt partially with LF regression after DAA therapy in HCV-infected patients^3–8,19^, to our knowledge our study has the longest follow-up and the second with the largest number of coinfected patients published so far in the English literature. Very recently Kronfli et al. reported , as we observed, progressive NILFM regression after 96 weeks of follow-up of 382 HCV-HIV coinfected patients after DAA therapy .However in this Canadian study only 149 of the patients were followed by TE and the rest by APRI and FIB-4 indexes^19^. The quick LF decline identified by non-invasive biochemical biomarkers, TE and plasma MMPs is due to early resolution of liver inflammation after clearance of HCV infection^2–7,40,41^. A recent Chinese study described persistent liver inflammation in HCV-infected patients with advanced LF after DAA-induced sustained viral response^8^, The authors observed that this persistence was associated with impaired liver function and recommended long-term follow-up of the patients. We did not find such persistent inflammation in the short- or long-term in our study, in spite of having enrolled 29.8% patients with the most advanced (F4) LF stage. In fact, quick reversion of the inflammation during the first month of therapy was responsible for the flattening of the curves of the three biochemical fibrosis biomarkers (APRI, Forns and FIB-4), which, consequently, proved to be useless for monitoring fibrosis during follow-up, a relevant conclusion of our study. On the other contrary, TE provided accurate monitoring of LF regression as long as 24 months after DAA therapy, confirming previous reports with shorter follow-ups^3–7,19^.

Plasma MMPs, especially TIMP-1, also experienced a downward course over time in parallel to TE, and baseline TIMP-1 significantly correlated with all the four noninvasive methods of fibrosis evaluation. In this regard, Boeker et al. reported that TIMP-1 and MMP-2 plasma levels correlated with biopsy LF changes in 59 German patients with chronic HCV infection and in 19 with HCV-induced liver cirrhosis^27^. Larrousse et al^14^. observed that baseline TIMP-1 was quite sensitive and specific for predicting the degree of LF in 119 HCV-HIV coinfected Spanish patients who did not undergo follow-up. Latronico et al. found that TIMP-1 plasma levels correlated well with LF in a small group of 16 mono- and 15 coinfected Italian patients^41^. However, TIMP-1 plasma levels did not change after therapy composed of pegylated interferon-α, ribavirin and HCV protease inhibitors (telaprevir or boceprevir). The very low number of patients, the short follow-up (3 months) and the use of a less effective treatment would explain this discrepancy with our findings. Leroy et al. reported than circulating MMP-1 was better than TIMP-1, MMP-2 and -9 to monitor biopsy-assessed LF in 194 HCV-infected French patients^28^. Neither the effect of anti-HCV therapy on LF nor a dynamic extended follow-up of circulating MMPs and TIMPs .were assessed in the previous study. El-Kamary et al. observed that a combination of 12 biochemical markers including TIMP-1, MMP-1 and MMP-2 had a high accuracy to diagnose the five stages of LF according to the METAVIR score in Egyptian patients with chronic HCV infection^29^. Finally, a recent study reported that the MMP9/TIMP1 ratio correlated well with LF assessed by noninvasive biochemical markers and TE in 33 HCV monoinfected Brazilian patients followed-up for only the 12 weeks of sofosbuvir-based DAA therapy^30^. In our much larger study the MMP-9/TMP-1 ratio was not superior to TIMP-1 as fibrosis indicator.

Interestingly, as opposed to the degree of fibrosis, we observed consistently higher levels of MMPs in coinfected than in monoinfected patients, but the opposite regarding the MMP inhibitor TIMP-1. In the small study of Latronico et al.^41^, the 15 coinfected patients had higher levels of MMP-9 than the 16 monoinfected patients, but there were no differences regarding MMP-2, MMP-8 and TIMP-1. This balance between the enzyme and the inhibitor might also explain the higher degrees of fibrosis that we observed in monoinfected as compared to coinfected patients.

However the greater variability of the MMPs, as well as and the different levels depending on the HIV status, limit to some extent the usefulness of a single measurement as a reliable marker of fibrosis in an individual patient, although sequential measurements of these proteins, particularly TIPM-1, could be more helpful for monitoring as a non-invasive marker the fibrosis course over time. The continuous decline of MMPs plasma levels throughout the follow-up suggests that a reduction of ECM remodeling and connective tissue proteins synthesis is the tissue correlate of these plasma findings, and that lasted at least two years after DAA therapy. A pathogenic mechanism that cannot be identified by the usual noninvasive biochemical markers or TE, reinforcing therefore the value of sequential measurements of plasma MMPs, especially of TIMP-1.

The strongest points of our study include its prospective nature, the very long follow-up after DAA therapy, the high number of patients enrolled and the comprehensive evaluation at each time point. However there are also some limitations, including the measurement of MMPs and TIMP-1 plasma levels in only a subset of the enrolled patients due to budget limitations, although the sample size was large enough to detect significant differences. Another limitation is the lack of liver biopsies, the gold standard for fibrosis. However, biopsies are invasive, have also limitations, are being replaced by TE and we used this technique along with other noninvasive biochemical markers of LF. Finally, the multiplicity and efficacy of DAA combinations preclude the evaluation of the effect of specific regimens, although this shortcoming does not affect the evaluation of the fibrosis response to DAA therapy.

We conclude that LF regression is a dynamic process that begins early after institution of DAA therapy and last for at least 24 months. This LF regression might be due to a reduction of ECM synthesis and connective-tissue remodeling slow-down, as reflected by MMPs and TIMP-1 plasma levels decline. The LF reversal is best monitored by TE and plasma TIMP-1 levels, because other laboratory-derived noninvasive markers are not valid beyond the first month, and the greatest absolute and relative improvements in NILFM are observed in patients with higher fibrosis stages at baseline. HCV-monoinfected patients have higher baseline NILFM than those coinfected with HCV-HIV, although the fibrosis response to therapy is similar, and have also lower levels of MMPs and higher levels of TIMP-1 than coinfected patients. Regarding DAA treatment, sofosbuvir-containing regimens have similar efficacy in NILFM reduction as sofosbuvir-free regimens.

## Patients and methods

### Patients

Patients with active HCV monoinfection or HCV-HIV coinfection demonstrated by positive serology and viral RNA plasma levels were enrolled in the study when starting DAA therapy. Patients were older than 18 years and were recruited from three third level hospitals of Northwest Spain. A number of demographic, epidemiological, laboratory and clinical data were obtained from the patients and from their electronic medical charts. All HCV-HIV-coinfected patients were receiving ART at the inclusion time. The DAA regimens used included NS5B inhibitors (sofosbuvir, dasabuvir), NS3/4A inhibitors (paritaprevir, asunaprevir, grazoprevir, simeprevir) and NS5A inhibitors (ledipasvir, daclatasvir, ombistavir, elbasvir) with or without ribavirin. Throughout the whole study, DAA treatment initiation was independent of fibrosis stage.

All patients were members of a homogenous Caucasian population and were residents in Northwest Spain (Asturias and Galicia), a region with a small foreign immigrant population. DAA were selected according the attending clinician criteria. LF was assessed by TE (Fibroscan) and by the noninvasive biochemical biomarkers APRI, Forns and FIB-4 at baseline and at the 1st, 3rd, 6th, 12th and 24th months. MMP -2, -8,-9 and TIMP-1 plasma levels were assessed in a subgroup of patients at the same time points.

All patients underwent standard of care, including routine noninvasive procedures, and signed an informed consent before inclusion in the study. This study was approved by the Ethics Committee of the Hospital Universitario Central de Asturias (HUCA). In addition all methods mentioned in this manuscript were performed in accordance with the relevant guidelines and regulations.

### Exclusion criteria

To avoid LF confounding factors different from HCV and HIV infections, patients with HBV coinfection with/out delta virus coinfection, ethanol consumption ≥ 50 g/d for > 5 years, alcoholic hepatopathy, and other liver diseases were excluded from the study as we previously did^38^. Pregnant women and those individuals in whom there were technical difficulties for obtaining reliable TE readings were also excluded. In addition, patients with ascites or spontaneous bacterial peritonitis were excluded because TE reading could be altered by these factors^15–18^.

### Transient elastometry

LF was assessed by TE using Fibroscan (EchoSens,Paris, France) following pre-established methods^15–18^. Liver stiffness was the median value of ≥ 10 measurements obtained from the right liver lobe through one or two right thoracic intercostal spaces, expressed in kPa. The success rate was calculated as the number of validated measurements divided by the total number of assessments. The median value of successful measurements was considered representative of liver stiffness only if the interquartile range (IQR) of all validated measurements was < 30% of the median value with a success rate > 60%. Patients were classified into 4 groups according to the TE measurements (F0-F1, F2, F3 and F4), which reflects the progressive degree of LF by similitude with the histological stages of the METAVIR grading system. Thus, < 7.2 kPa measurements were considered minimal or no fibrosis (F0-F1), values in the range of 7.2–9.3 kPa were considered as indicative of significant fibrosis (F2), those in the range of 9.4–13.9 kPa advanced fibrosis (F3), and values > 13.9 kPa were considered cirrhosis (F4).

### Laboratory methods

HIV and HCV serologies were assessed by enzyme immunoassay (MEIA AxSYM; Abbott Diagnostics,Abbott Park, IL, United States). HIV and HCV RNA by quantitative PCR (Cobas TaqMan; Roche Diagnostics, Branchburg, NJ, United States) and HCV genotypes by a line probe assay (Versant HCV, Siemens). Routine laboratory methods were used to calculate three LF indexes: aspartate aminotransferase (AST) and platelets for APRI index^42^, age, platelet counts, total cholesterol and GGT for Forns index^43^, and age, AST, alanine amino transferase (ALT) and platelet counts for FIB-4^44^.

### MMPs and TIMPs plasma levels assessment

Ten milliliters of whole blood were collected in glass tubes containing potassium-EDTA, and centrifuged at 1800 × g for 5 min. Then the obtained plasma was aliquoted in Eppendorf tubes and stored at − 80 ℃ until further use. MMPs (-2, -8, -9) and TIMP-1 were measured individually using an ELISA sandwhich technique (FineTest, Wuhan Fine Biotech Co. Wuhan, China), according to the manufacturer’s instructions. Due to budget limitations plasma MMPs and TIMP-1 were only measured in 61 patients. Each patient was given an ordinal number after study enrollment. Considering the budget limitations the last out of every six patients was selected for having his/her MMPs and TIMPs plasma levels assessed* .* Selected individuals were equally distributed regarding age, sex, HCV mono/HCV-HIV coinfection, HCV genotype and DAA therapy administered (with/out sofosbuvir).

### MMPs SNPs genotyping

DNA was obtained from peripheral white blood cells and stored at -20 ℃ until use. The following SNPs of MMPs were genotyped using the StepOne real‐time PCR system with TaqMan 5′‐exonuclease allelic discrimination assays (Assay‐on‐Demand service, Applied Biosystems, Foster City, California): *MMP-2 (-1306 C/T*, rs 243865 and *-735 C/T,* rs2285053), *MMP-8(-799 C/T, rs 11225395*), *MMP-13(-77 A/G, rs 2252070)* and *TIMP-1 (-9830 T/G, rs 2070584).* The primers and probe sequences were obtained from the National Cancer Institute SNP500 database. The probes were fluorescently labeled with either FAM or VIC dyes on the 5′ end and a nonfluorescent minor groove binder quencher on the 3′ end (Applied Biosystems) as previously published^45^.

### Statistical analysis

As the distribution of MMPs, TIMP-1 and fibrosis parameters was not Gaussian, they underwent natural logarithmic transformation for analysis and were back-transformed into the original units for reporting. Categorical variables are described as percentage and were compared by the chi-square and Fisher’s exact tests, as appropriate. Continuous variables are described as mean and 95% confidence intervals and were assessed by the t-test, in the case of two groups, and one-way ANOVA for more than two groups. The Pearson correlation coefficient was used to evaluate the relationships between MMPs, TIMP-1 and fibrosis parameters. Paired t-tests were used to compare intra-subject changes in the four fibrosis indexes between two different time points. The factors independently associated with the mono/coinfected status were identified by means of a stepwise logistic regression analysis and the factors related to improvement in LF during follow-up were identified by backward multiple regressions. Statistical analyses were carried out with the SPSS v.25 software (IBM Corp., Armonk, NY, USA). A *P* < 0.05 level for a two-tailed test was considered statistically significant.

## Supplementary Information

Supplementary Information 1.

Supplementary Information 2.
