# Supplementary material for: 24-month decline of non-invasive liver fibrosis markers in HCV-mono and HCV/HIV coinfection after direct-acting antiviral therapy
Source: Sci Rep. 2022 Mar 9;12:3828. doi: 10.1038/s41598-022-07548-y (PMC8907337; doi:10.1038/s41598-022-07548-y)
Supplement: Supplementary file 1 — Supplementary Information 1. [file 41598_2022_7548_MOESM1_ESM.doc]

Fig.S1 (supplementary). Course over time of the four fibrosis indexes according to the *MMP-2 (-1306C/T****)*** genotypes (mean, 95% CI).
