# Supplementary figures and images for: 24-month decline of non-invasive liver fibrosis markers in HCV-mono and HCV/HIV coinfection after direct-acting antiviral therapy
Source: Sci Rep. 2022 Mar 9;12:3828. doi: 10.1038/s41598-022-07548-y (PMC8907337; doi:10.1038/s41598-022-07548-y)

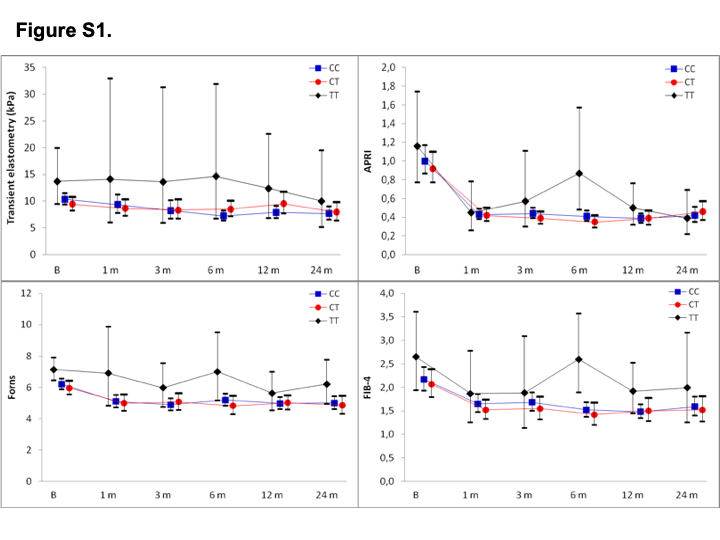

Supplement: Supplementary file 2 — Supplementary Information 2. [file 41598_2022_7548_MOESM2_ESM.tiff]
